# Supplementary material for: Identification of Genes Associated with Gut Microbiota Metabolites in Type 2 Diabetes Mellitus Complicated by Acute Cerebral Infarction Based on Bioinformatics and Mendelian Randomization
Source: Biomedicines. 2026 Oct 17;14(10):2371. doi: 10.3390/biomedicines14102371 (PMC13702449; doi:10.3390/biomedicines14102371)
Supplement: Supplementary file 1 [file biomedicines-14-02371-s001.zip › Figure S2/class.Erysipelotrichia.id.2147 T2DM.pdf]

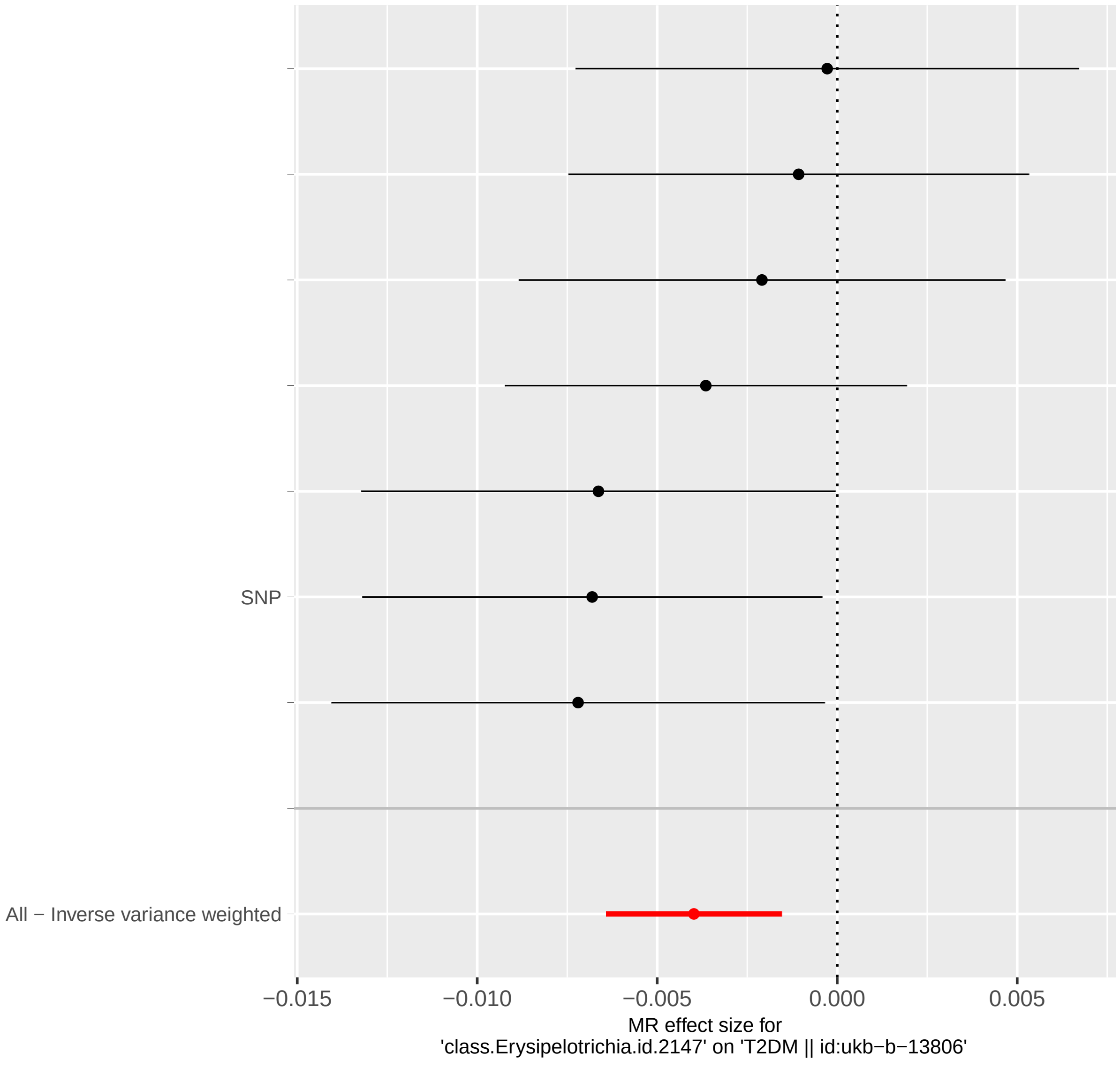
SNP
All − Inverse variance weighted
−0.015
−0.010
−0.005
0.000
0.005
MR effect size for
'class.Erysipelotrichia.id.2147' on 'T2DM || id:ukb−b−13806'
